# Co-exposure to lipopolysaccharide and desert dust causes exacerbation of ovalbumin-induced allergic lung inflammation in mice via TLR4/MyD88-dependent and -independent pathways

**DOI:** 10.1186/s13223-019-0396-4

**Published:** 2019-12-18

**Authors:** Yahao Ren, Takamichi Ichinose, Miao He, Seiichi Youshida, Masataka Nishikawa, Guifan Sun

**Affiliations:** 1 Department of Nutritional and Food Hygiene, School of Public Health, China Medical University, Shenyang, 110122 China; 2 Department of Health Sciences, Oita University of Nursing and Health Sciences, Oita, 870-1201 Japan; 3 Department of Environmental Health, School of Public Health, China Medical University, Shenyang, 110122 China; 4 Environmental Chemistry Division, National Institute for Environmental Studies, Ibaraki, 305-8506 Japan

**Keywords:** Lipopolysaccharide, Airway-inflammation, TLR4, MyD88, TRIF

## Abstract

**Background:**

Lipopolysaccharide (LPS) often presents in high concentrations in particulate matter (PM), few studies have reported the enhancing effects of both LPS and PM on airway inflammation in mice and the role of toll-like receptors (TLRs) in this process. Asian sand dust (ASD) is observed most frequently during the spring. This study aimed to clarify the role of TLRs in murine lung eosinophilia exacerbated by ASD and LPS.

**Methods:**

The effects of LPS and ASD co-treatment on ovalbumin (OVA)-induced lung eosinophilia were investigated using wild-type (WT), TLR2^−/−^, TLR4^−/−^, and adaptor protein myeloid differentiation factor 88 (MyD88)^−/−^ BALB/c mice. ASD was heated (H-ASD) to remove the toxic organic substances. WT, TLR2^−/−^, TLR4^−/−^ and MyD88^−/−^ BALB/c mice were intratracheally instilled with four different combinations of LPS, H-ASD and OVA treatment. Subsequently, the pathological changes in lungs, immune cell profiles in bronchoalveolar lavage fluid (BALF), inflammatory cytokines/chemokines levels in BALF and OVA-specific immunoglobulin (Ig) in serum were analyzed.

**Results:**

In WT mice, H-ASD + LPS exacerbated OVA-induced lung eosinophilia. This combination of treatments increased the proportion of eosinophils and the levels of IL-5, IL-13, eotaxin in BALF, as well as the production of OVA-specific IgE and IgG1 in serum compared to OVA treatment alone. Although these effects were stronger in TLR2^−/−^ mice than in TLR4^−/−^ mice, the expression levels of IL-5, IL-13, eotaxin were somewhat increased in TLR4^−/−^ mice treated with OVA + H-ASD + LPS. In MyD88^−/−^ mice, this pro-inflammatory mediator-induced airway inflammation was considerably weak and the pathological changes in lungs were negligible.

**Conclusions:**

These results suggest that LPS and H-ASD activate OVA-induced Th2 response in mice, and exacerbate lung eosinophilia via TLR4/MyD88, TLR4/TRIF and other TLR4-independent pathways.

## Background

Lipopolysaccharide (LPS), a cell wall component of gram-negative bacteria, is ubiquitous in nature. Epidemiological studies have demonstrated that LPS exposure is a significant risk factor for increased asthma prevalence [1, 2]. LPS often presents in high concentrations in particulate matter (PM) [3]. Asian sand dust (ASD) is observed most frequently during the spring. When a massive sandstorm occurs in Northern China and Mongolia, ASD can be spread by aerosols over large geographic areas [4]. Epidemiological studies suggest that ASD events are associated with increased mortality rates due to respiratory and circulatory diseases [5, 6].

LPS is often found in significantly higher concentrations in ASD [7]. Our previous study showed that 1 and 10 ng of LPS exacerbated the effect of heated-ASD (H-ASD) on ovalbumin (OVA)-induced lung eosinophilia [8]. Another study has shown that low-dose LPS (100 ng) facilitates an allergen-specific Th2 response, while high-dose LPS (10 μg) induces a Th1 response [9], but the underlying mechanism remains largely unclear. A wide panel of signal transduction pathways is activated by LPS, in which Piggott and colleagues have reported that low-dose LPS (8–120 ng) activate Th2 responses to OVA through myeloid differentiation factor 88 (MyD88) protein [10]. However, we sought to increase the amount of LPS in order to clarify the mechanism underlying the exacerbative effect of LPS + ASD, as it is believed that the exacerbation pathway can be complemented by increasing LPS dose.

Toll-like receptors (TLRs) are pattern recognition receptors (PRRs) that play an essential role in animal immunity [11]. TLR4 has been long recognized as a receptor for LPS, which activates two signaling pathways: TLR4/MyD88 pathway and TLR4/TIR-domain-containing adapter-inducing interferon-β (TRIF) pathway [12, 13].

In the present study, the role of TLRs in the exacerbation of lung eosinophilia was elucidated by exposing PM and LPS (50 ng) to mice. Specifically, the exacerbative effects of LPS and H-ASD on OVA-induced lung eosinophilia were investigated using wild-type (WT), TLR2^−/−^, TLR4^−/−^ and MyD88^−/−^ mice with BALB/c background.

## Methods

### Mice

Specific pathogen-free male WT, TLR2^−/−^, TLR4^−/−^ and MyD88^−/−^ (on BALB/c background, 6 weeks of age) were obtained from Charles River Japan, Inc. (Kanagawa, Japan). The body weight of the mice and the presence of infection were checked for 1 week. The mice used were 7 weeks of age. CE-2 commercial diet (CLEA Japan, Tokyo, Japan) and water were given ad libitum. The mice were housed in plastic cages lined with soft wood chips. The cages were placed in a conventional room, which was air conditioned at 23 °C with a light/dark (12 h/12 h) cycle, and humidity ranging from 55 to 70%. The study adhered to the US National Institutes of Health guidelines for the use of experimental animals. The animal care method was also approved by the animal care and use committee at Oita University of Nursing and Health Sciences in Oita, Japan.

### Preparation of particles and LPS

The ASD used as the standard base for the samples in this study was collected from surface soils in the Gobi desert and purified for use in the present study. The size distribution peak was observed at 3.9 µm. The chemical elements in ASD were as reported previously: 51.6% SiO_2_, 14.3% Al_2_O_3_, 5.5% Fe_2_O_3_, 1.3% Na_2_O, 9.6% CaCO_3_, 0.6% CaO, 2.5% MgO, 0.7% TiO_2_ and 2.6% K_2_O. And, as in the previous study, remaining 11.3% was included as other oxides [14]. A portion of the standard ASD was heated at 360 °C for 30 min in an electric heater to exclude toxic materials (sulfate, nitrate, microorganism, etc.). These samples are termed heated-ASD (H-ASD) in the present study. Ultra pure LPS was purchased from InvivoGen (San Diego, CA, USA).

### Study protocol

One hundred and eight WT, TLR2^−/−^, TLR 4^−/−^ and MyD88^−/−^ male mice (on BALB/c background) were divided into six groups (n = 4 or 5 per group) separately, and each group was treated with a specific testing sample. The six testing samples (0.1 ml each of 0.9% NaCl normal saline solution) prepared for the present study were control (containing normal saline alone); LPS (50 ng LPS); H-ASD (0.1 mg H-ASD); OVA (2 µg OVA alone); OVA + LPS (2 µg OVA and 50 ng LPS); OVA + H-ASD + LPS (2 µg OVA, 0.1 mg H-ASD, and 50 ng LPS). The mice were intratracheally exposed to a mixed or individual solution of OVA, H-ASD and LPS 4 times at 2-week intervals. The control group was instilled intratracheally with 0.1 ml normal saline.

### Bronchoalveolar lavage fluid (BALF)

All mice were used for an examination of the free cell contents in BALF. BALF and cell counts were conducted using a previously reported method [8, 15]. Briefly, the lungs were lavaged with two injections of 0.8 ml of sterile saline at 37 °C. After the fluids from the two lavages were combined and cooled to 4 °C, the resultant solution was centrifuged at 1500 rpm for 10 min. The protein levels of cytokines and chemokines in the BALF were measured. The total cell count of the fresh fluid specimen was determined by a hemocytometer. Differential cell counts were assessed on cytological preparations. Slides were prepared using a Cytospin (Sakura Co., Ltd, Tokyo, Japan) and stained with Diff-Quik (International Reagents Co., Kobe, Japan) to identify the eosinophils with red granules. A total of 300 cells were counted under a microscope. The BALF supernatants were stored at − 80 °C to await analysis for cytokines and chemokines.

### Pathological evaluation

All mice were used for pathological examination. The lungs were fixed by 10% neutral phosphate-buffered formalin. After separation of the lobes, 2-mm-thick blocks were taken for paraffin embedding. Embedded blocks were sectioned at a thickness of 3 µm, and then stained with May-Grunwald’s stain solution (Nacalai tesque, Inc, Kyoto, Japan) and Giemsa’s azur eosine methylene blue solution (Merck KGaA, Darmstadt, Germany) to evaluate the degree of infiltration of eosinophils and lymphocytes in the airway from proximal to distal. The sections were stained with periodic acid-Schiff (PAS) to evaluate the degree of proliferation of goblet cells in the bronchial epithelium. A pathological analysis of inflammatory cells and epithelial cells in the airway was performed using a Nikon ECLIPSE light microscope (Nikon Co., Tokyo, Japan). The degree of infiltration of eosinophils and lymphocytes in the airway or proliferation of goblet cells in the bronchial epithelium was graded in a blinded fashion: 0, not present; 1, slight; 2, mild; 3, moderate; 4, moderate to marked; 5, marked. ‘Slight’ was defined as less than 20% of the airway with eosinophilic inflammatory reaction or with goblet cells stained with PAS; ‘mild’ as 21–40%; ‘moderate’ as 41–60%; ‘moderate to marked’ as 61–80%; and marked as more than 80% of the airway [8, 16].

### Quantitation of cytokines and chemokines in BALF

The cytokine and chemokine protein levels were determined by enzyme-linked immunosorbent assays (ELISA). IL-5 and IL-12 were measured using an ELISA kit from Endogen, Inc. (Cambridge, MA, USA). Monocyte chemotactic protein (MCP)-3 was measured using an ELISA kit from Bender MedSystems Inc. (Burlingame, CA, USA). IL-1β, IL-6, IL-13, IL-17A, Interferon (IFN)-γ, Keratinocyte chemoattractant (KC), Tumor necrosis factor (TNF)-α, Transforming growth factor (TGF)-β, eotaxin, MCP-1, were measured using an ELISA kit from R&D Systems Inc. (Minneapolis, MN, USA).

### OVA-specific IgE and IgG1 antibodies

OVA-specific immunoglobulin E (IgE) and IgG1 antibodies were measured using the Mouse OVA-IgE ELISA kit and Mouse OVA-IgG1 ELISA kit (Shibayagi Co., Shibukawa, Japan). According to the manufacturer’s protocol, 1U of the anti-OVA IgE is defined as 1.3 ng of the antibody; and 1U of the anti-OVA IgG1 is defined as 160 ng of the antibody. The absorption of 450 nm (sub-wave length, 620 nm) for OVA-specific IgE and IgG1 antibody was measured by a microplate reader (Spectrafluor, Tecan, Salzburg, Austria).

### Statistics

Statistical analysis on the pathological evaluation in the airway, cytokines, and chemokine proteins in BALF were conducted using one-way ANOVA with post hoc Tukey test. All the analyses were performed with IBM SPSS Statistics Client21 (AsiaAnalytics, Shanghai, China). Differences among groups were determined as statistically significant at a level of *P* < 0.05.

## Results

### Inflammatory cell profile changes in the BALF of mice treated by OVA, H-ASD and LPS

**Fig. 1:**
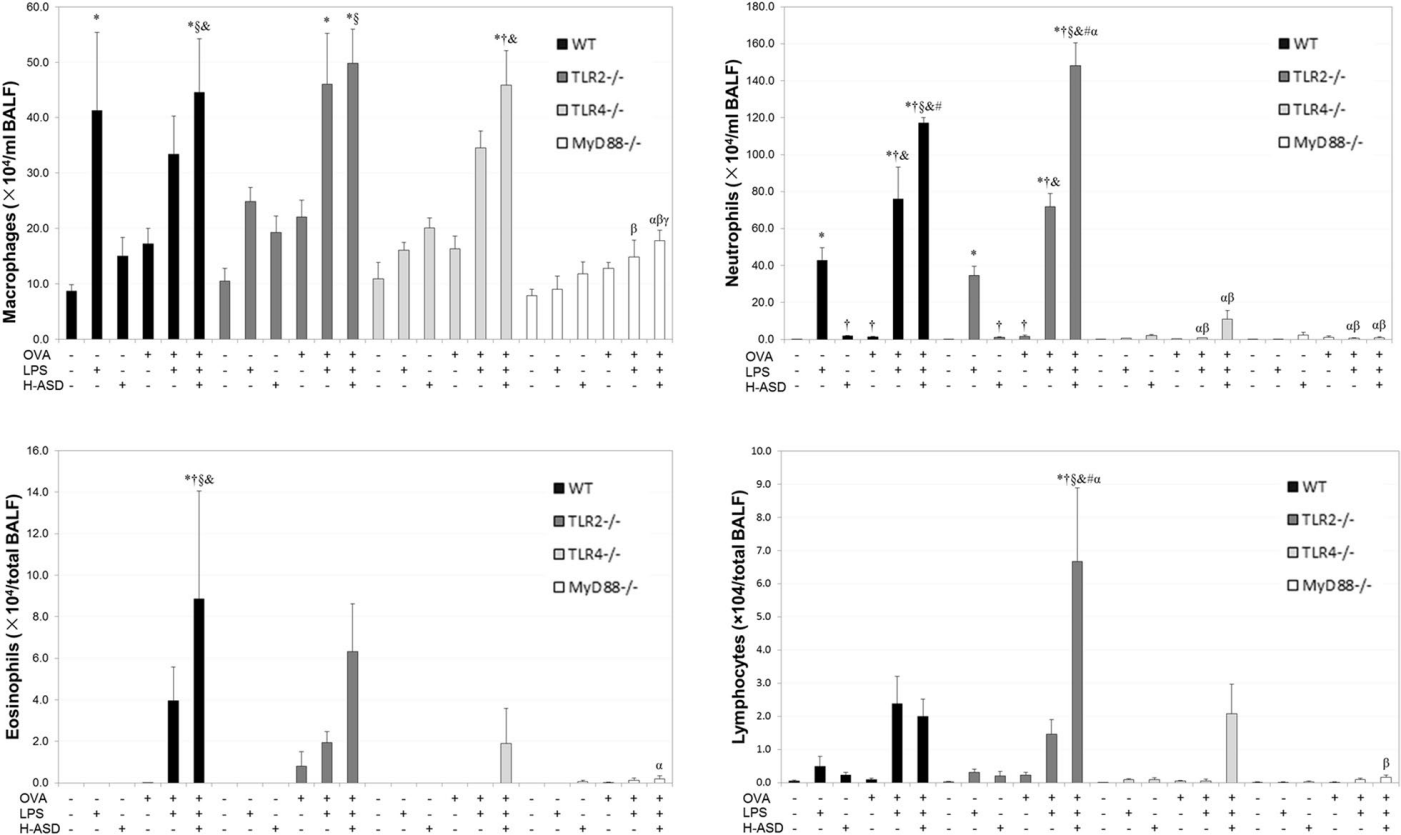
Immune cell profile changes in bronchoalveolar lavage fluid (BALF) of mice. Wild type (WT), toll like receptor (TLR)2^−/−^, TLR4^−/−^, myeloid differentiation primary response gene 88 (MyD88)^−/−^, four kinds of mouse were divided into six groups which were treated intratracheally with saline (control), LPS (50 ng LPS); heated-Asian sand dust (H-ASD) (0.1 mg H-ASD); ovalbumin (OVA), OVA + lipopolysaccharide (LPS), OVA + H-ASD + LPS. Four times at 2-week intervals. All values were expressed as mean ± SEM. *P < 0.05 vs. control; ^†^*P* < 0.05 vs. LPS; ^§^*P* < 0.05 vs. H-ASD; ^&^*P* < 0.05 vs. OVA; ^#^*P* < 0.05 vs. OVA + LPS; ^α^*P* < 0.05 vs. WT within the same group; ^β^*P* < 0.05 vs. TLR2^−/−^ within the same group; ^γ^*P* < 0.05 vs. TLR4^−/−^ within the same group

Figure 1 shows the immune cell profiles in BALF. Compared to saline group, LPS significantly increased the proportion of macrophages and neutrophils, and OVA + LPS further increased the proportion of neutrophils in WT mice. In addition, OVA + H-ASD + LPS increased significantly the proportions of macrophages, neutrophils and eosinophils compared to saline and OVA groups. The changes in the distributions of macrophages, neutrophils, eosinophils and lymphocytes in TLR2^−/−^ mice were relatively similar to those in WT mice, while the change in macrophage distribution in TLR4^−/−^ mice were also similar to that in WT mice. However, the numbers of these inflammatory cells were reduced by OVA + H-ASD + LPS treatment in MyD88^−/−^ mice compared to WT and TLR2^−/−^ mice.

### Pathological changes in the airways of mice treated by OVA, H-ASD and LPS

**Fig. 2:**
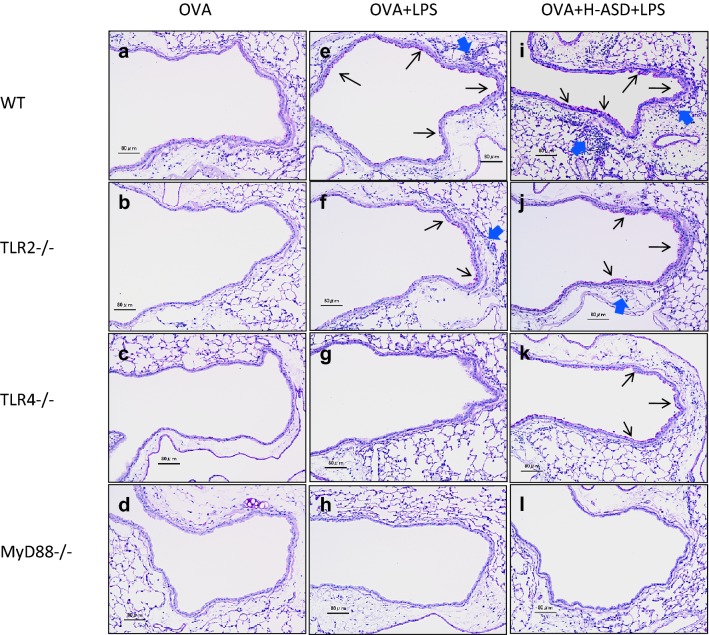
Effects of testing samples on pathological changes in the lungs. **a**–**l** PAS stain; Bar = 80 μm. **a** WT ovalbumin (OVA); **b** TLR2^−/−^ OVA; **c** TLR4^−/−^ OVA; **d** MyD88^−/−^ OVA: no pathological changes in lungs treated with OVA. **e** WT OVA + lipopolysaccharide (LPS); **f** TLR2^−/−^ OVA + LPS: very slight proliferation of goblet cells (thin arrow) that have mucus stained pink with PAS in the airway epithelium, and peribronchiolar inflammation due to slight infiltration of inflammatory cells into the submucosa of airways. **g** TLR4^−/−^ OVA + LPS; **h** MyD88^−/−^ OVA + LPS: no pathological changes in lungs treated with OVA + LPS. **i** WT OVA + heated-Asian sand dust (H-ASD) + LPS: moderate proliferation of goblet cells (thin arrow) in the airway epithelium, and moderate to marked infiltration of inflammatory cells (arrow) into the submucosa of airways. **j** TLR2^−/−^ OVA + H-ASD + LPS: slight to moderate proliferation of goblet cells (thin arrow) in the airway epithelium, and slight infiltration of inflammatory cells (arrow) into the submucosa of airways. **k** TLR4^−/−^ OVA + H-ASD + LPS: very slight proliferation of goblet cells (thin arrow) in the airway epithelium, and very slight peribronchiolar inflammation (arrow). **l** MyD88^−/−^ OVA + H-ASD + LPS: no significant pathological changes in airway epithelium or airway submucosa

**Fig. 3:**
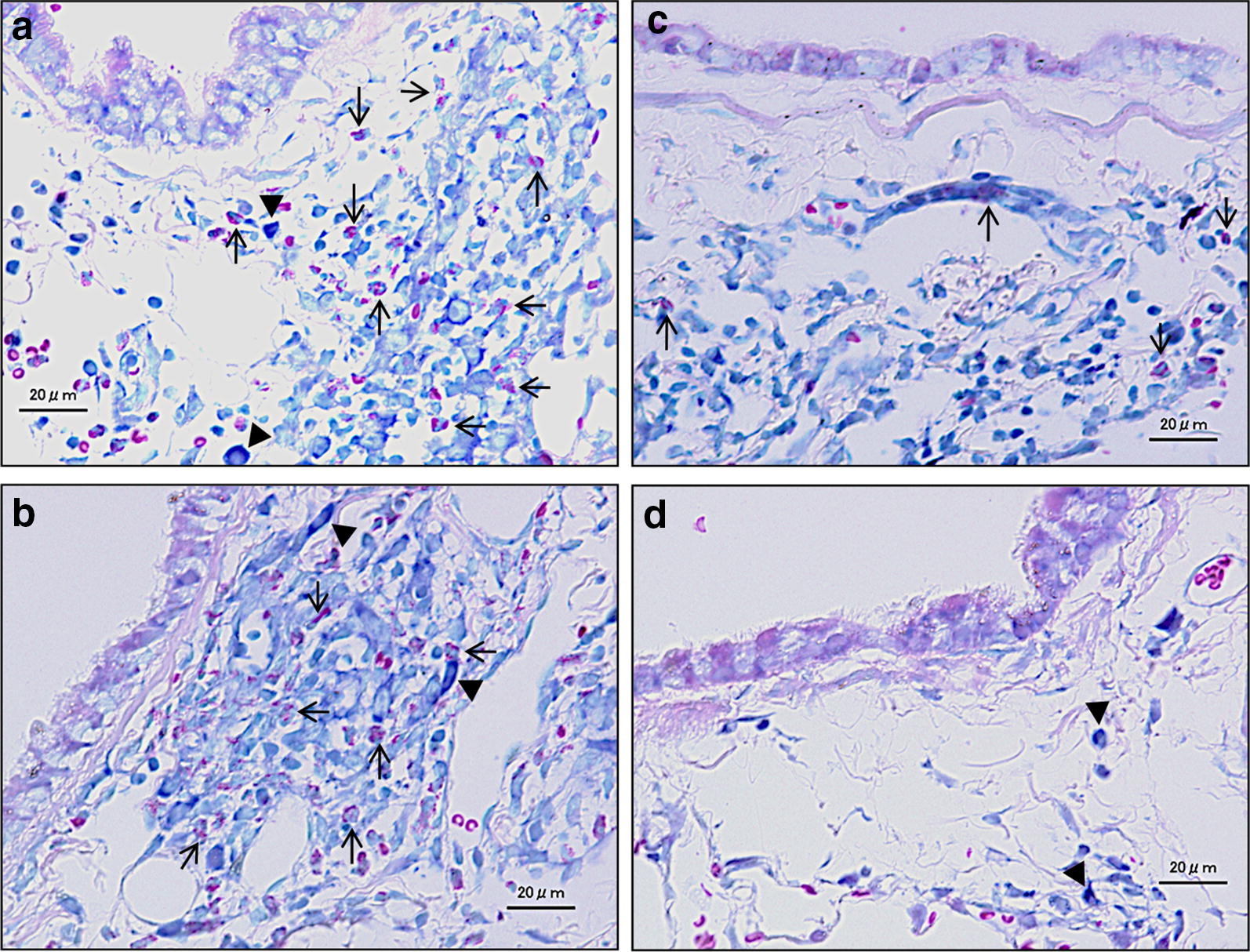
Effects of testing samples on infiltration of inflammatory cells in the airway. **a**–**d** May-giemsa stain; bar = 20 μm. **a** WT ovalbumin (OVA) + heated-Asian sand dust (H-ASD) + lipopolysaccharide (LPS): moderate infiltration of eosinophils into the airway submucosa. **b** TLR2^−/−^ OVA + H-ASD + LPS: moderate infiltration of eosinophils. **c** TLR4^−/−^ OVA + H-ASD + LPS: slight infiltration of eosinophils. **d** MyD88^−/−^ OVA + H-ASD + LPS: no significant pathological changes in the airway submucosa. Arrows show eosinophils with red granules. Triangles show tissue macrophages

Figures 2 and 3 illustrate the pathological changes induced by OVA, H-ASD and LPS treatment in the murine airway. No pathological alterations were found in the lungs between the control and OVA groups (Fig. 2a–d). OVA + LPS slightly increased the proliferation of goblet cells in the airway epithelium, and slightly accelerated the infiltration of inflammatory cells in the airway submucosa of WT and TLR2^−/−^ mice (Fig. 2e, f). However, no pathological alterations were found in the lungs of TLR4^−/−^ and MyD88^−/−^ mice (Fig. 2g, h).

OVA + H-ASD + LPS induced mild to moderate proliferation of goblet cells in the airway epithelium, and slight to marked infiltration of inflammatory cells in the airway submucosa of WT and TLR2^−/−^ mice (Figs. 2i, j, 3a, b). Meanwhile, there was only very mild goblet cell proliferation observed in the airway epithelium, and very slight inflammatory cell infiltration in the airway submucosa of TLR4^−/−^ mice (Figs. 2k and 3c). Besides, no pathological alterations were found in the lungs of MyD88^−/−^ mice (Figs. 2l and 3d).

**Fig. 4:**
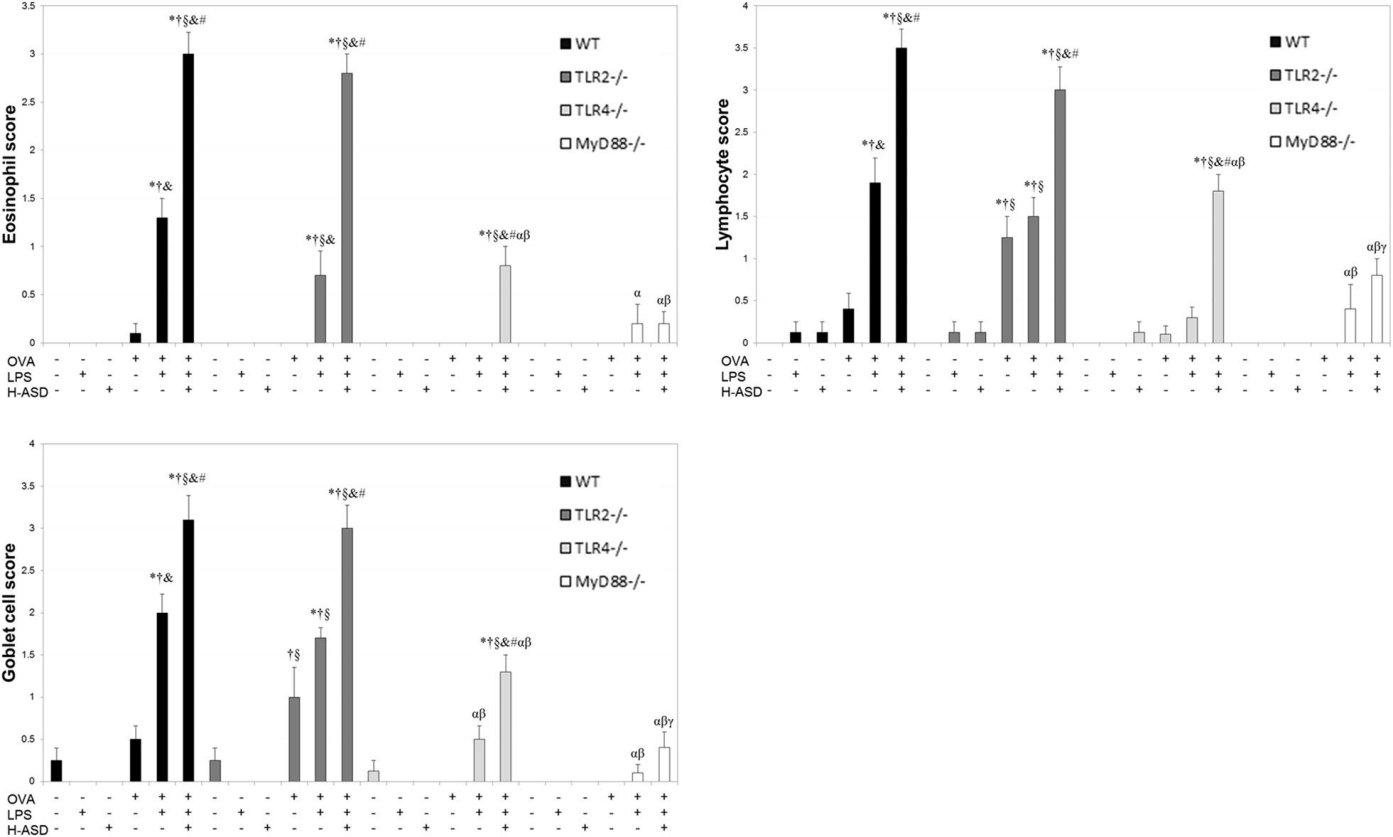
Evaluation of pathological changes in the murine airway. The degree of pathological changes in the airway was estimated as: (0) none; (1) slight; (2) mild; (3) moderate; (4) moderate to marked; (5) marked. Wild type (WT), toll like receptor (TLR)2^−/−^, TLR4^−/−^, myeloid differentiation primary response gene 88 (MyD88)^−/−^, four kinds of mouse were divided into six groups which were treated intratracheally with saline (control), LPS (50 ng LPS); heated-Asian sand dust (H-ASD) (0.1 mg H-ASD); ovalbumin (OVA), OVA + lipopolysaccharide (LPS), OVA + H-ASD + LPS. Four times at 2-week intervals. All values were expressed as mean ± SEM. *P < 0.05 vs. control; ^†^*P* < 0.05 vs. LPS; ^§^*P* < 0.05 vs. H-ASD; ^&^*P* < 0.05 vs. OVA; ^#^*P* < 0.05 vs. OVA + LPS; ^α^*P* < 0.05 vs. WT within the same group; ^β^*P* < 0.05 vs. TLR2^−/−^ within the same group; ^γ^*P* < 0.05 vs. TLR4^−/−^ within the same group

Figure 4 shows the abnormal cell changes caused by the indicated treatments in the murine airway. The rates of goblet cell proliferation and inflammatory cell infiltration among the four groups were increased in the following order: WT mice > TLR2^−/−^ mice > TLR4^−/−^ mice > MyD88^−/−^ mice.

### Protein expression levels of cytokines and chemokines in the BALF of mice treated by OVA, H-ASD and LPS

Table 1 and 2 demonstrate the levels of IL-1β, IL-5, IL-6, IL-12, IL-13, IL-17A, KC, MCP-1, MCP-3, IFN-γ, eotaxin and TNF-α in BALF. In WT mice, the baseline levels of these proteins in untreated samples were relatively low, LPS significantly increased IL-12 and TNF-α levels, H-ASD significantly increased KC levels, OVA slightly increased IL-1β, KC, IL-12, IL-13 and IFN-γ levels, the addition of LPS to OVA further upregulated the expression levels of all proteins, and OVA + H-ASD + LPS group exhibited the highest levels of all proteins. The changes in the expression levels of these proteins in TLR2^−/−^ mice were similar to those in WT mice. However, the expression levels of these proteins were decreased in TLR4^−/−^ and MyD88^−/−^ mice compared to WT and TLR2^−/−^ mice. Nevertheless, TGF-β level was not detected in the present study.

**Table 1:** Expression of cytokines in bronchoalveolar lavage fluid (pg/ml)

Group	Animals (n)	IL-1β	IL-5	IL-6	IL-12	IL-13	IL-17A
WT mice							
Control	4	0.1 ± 0.1	1.1 ± 0.3	nd	14.4 ± 1.5	nd	nd
LPS	4	4.5 ± 0.4	2.6 ± 0.8	17.8 ± 4.4	427.5 ± 74.6*	nd	nd
H-ASD	4	5.0 ± 1.4	0.7 ± 0.1	nd	111.8 ± 10.2^†^	nd	nd
OVA	5	5.0 ± 1.0	2.3 ± 0.8	nd	32.3 ± 4.4^†^	1.5 ± 0.3	nd
OVA + LPS	5	9.4 ± 0.7	51.0 ± 12.8	9.8 ± 2.2	185.3 ± 30.9*^†&^	6.1 ± 1.8	5.0 ± 1.8
OVA + H-ASD + LPS	5	12.5 ± 0.9*	113.0 ± 15.5*^†§&#^	31.1 ± 8.0*^§&#^	224.3 ± 49.4*^†&^	20.3 ± 3.7*^†§&#^	14.0 ± 3.2*^†§&#^
TLR2^−/−^ mice							
Control	4	0.4 ± 0.4	0.7 ± 0.2	nd	18.6 ± 4.6	nd	nd
LPS	4	2.7 ± 0.3	1.1 ± 0.3	14.4 ± 3.2	479.5 ± 39.0*	nd	nd
H-ASD	4	4.5 ± 0.8	0.8 ± 0.2	nd	127.8 ± 22.4^†^	nd	nd
OVA	5	3.7 ± 0.7	13.2 ± 10.0	0.5 ± 0.5	47.2 ± 17.9^†^	2.6 ± 2.0	nd
OVA + LPS	5	24.5 ± 6.4*^†&α^	44.5 ± 12.2	28.0 ± 7.7*^&α^	327.6 ± 48.7*^†&α^	6.8 ± 1.7	1.2 ± 0.7
OVA + H-ASD + LPS	5	15.0 ± 1.4*^†§&^	105.0 ± 19.9*^†§&#^	20.8 ± 6.3*^§&^	299.3 ± 39.2*^†§&^	14.8 ± 2.6*^†§&#^	13.4 ± 2.2*^†§&#^
TLR4^−/−^ mice							
Control	4	0.2 ± 0.1	0.6 ± 0.2	nd	30.1 ± 2.5	nd	nd
LPS	4	0.2 ± 0.2	1.7 ± 0.3	nd	22.1 ± 5.3	nd	nd
H-ASD	4	3.7 ± 1.1	1.6 ± 0.2	0.8 ± 0.5	155.9 ± 15.3	nd	nd
OVA	5	2.1 ± 0.1	3.7 ± 2.0	nd	24.4 ± 4.4	0.9 ± 0.3	nd
OVA + LPS	5	2.6 ± 0.8^β^	2.5 ± 1.0	nd^β^	25.4 ± 3.3^αβ^	1.6 ± 0.7	nd
OVA + H-ASD + LPS	5	3.9 ± 0.8^β^	38.7 ± 25.7^αβ^	4.6 ± 2.9^α^	81.1 ± 21.3^αβ^	3.9 ± 2.4^αβ^	nd^αβ^
MyD88^−/−^ mice							
Control	4	1.0 ± 0.3	0.2 ± 0.1	nd	5.0 ± 0.5	nd	nd
LPS	4	1.4 ± 0.3	0.7 ± 0.4	nd	12.1 ± 1.9	nd	nd
H-ASD	4	8.4 ± 3.8	1.3 ± 0.4	3.7 ± 3.1	42.4 ± 8.7	0.2 ± 0.2	nd
OVA	5	7.2 ± 2.3	1.2 ± 0.1	nd	6.0 ± 0.6	1.1 ± 0.3	nd
OVA + LPS	5	8.4 ± 2.5^β^	14.4 ± 12.5	nd^β^	22.2 ± 2.1^αβ^	2.7 ± 1.6	nd
OVA + H-ASD + LPS	5	6.0 ± 1.2	10.1 ± 5.3^αβ^	0.6 ± 0.6^αβ^	83.2 ± 7.8^αβ^	3.4 ± 1.6^αβ^	nd^αβ^

Wild type (WT), toll like receptor (TLR)2^−/−^, TLR4^−/−^, myeloid differentiation primary response gene 88 (MyD88)^−/−^, four kinds of mouse were divided into six groups with 4–5 (n) which were treated intratracheally with saline (Control), lipopolysaccharide (LPS), heated-Asian sand dust (H-ASD), ovalbumin (OVA), OVA + LPS, OVA + H-ASD + LPS. Four times at 2-week intervals. All values were expressed as mean ± SEM

*nd* not detected

*p < 0.05 vs. control; ^†^*p* < 0.05 vs. LPS; ^§^*p* < 0.05 vs. H-ASD; ^&^*p* < 0.05 vs. OVA; ^#^*p* < 0.05 vs. OVA + LPS; ^α^*p* < 0.05 vs. WT within the same group; ^β^*p* < 0.05 vs. TLR2^−/−^ within the same group

**Table 2:** Expression of chemokines in bronchoalveolar lavage fluid (pg/ml)

Group	Animals (n)	IFN-γ	KC	MCP-1	MCP-3	Eotaxin	TNF-α
WT mice							
Control	4	nd	38.5 ± 8.6	nd	4.0 ± 0.5	1.4 ± 0.1	0.7 ± 0.1
LPS	4	nd	54.9 ± 10.4	13.2 ± 4.1	8.0 ± 1.4	5.5 ± 1.8	19.1 ± 5.4*
H-ASD	4	nd	183.6 ± 9.9*^†^	48.4 ± 7.4	7.5 ± 0.3	1.5 ± 0.1	4.6 ± 0.3
OVA	5	0.4 ± 0.1	53.6 ± 5.9^§^	1.8 ± 0.5	3.9 ± 0.6	1.3 ± 0.2	nd^†^
OVA + LPS	5	1.5 ± 0.6	113.4 ± 15.1*^§&^	26.4 ± 6.4	13.0 ± 1.8	16.1 ± 4.2*^†§&^	16.2 ± 1.9*^&^
OVA + H-ASD + LPS	5	1.9 ± 0.5*^†§^	147.3 ± 25.2*^†&^	129.8 ± 30.9*^†&#^	54.3 ± 20.2*^†§&#^	26.9 ± 4.4*^†§&#^	20.1 ± 5.0*^§&^
TLR2^−/−^ mice							
Control	4	nd	57.1 ± 8.3	nd	3.7 ± 0.3	1.4 ± 0.1	0.7 ± 0.1
LPS	4	nd	81.3 ± 10.5	16.9 ± 3.2	7.5 ± 0.6	4.1 ± 0.6	12.7 ± 2.8
H-ASD	4	nd	162.0 ± 19.2*^†^	54.2 ± 10.7	8.8 ± 0.6	1.5 ± 0.2	4.1 ± 0.7
OVA	5	0.5 ± 0.3	40.8 ± 20.6^§^	1.5 ± 1.4	4.3 ± 1.0	1.1 ± 0.5	nd
OVA + LPS	5	3.1 ± 0.8*^†&^	149.8 ± 12.4*^†&^	36.9 ± 8.8	16.0 ± 1.3	14.9 ± 2.1*^†&^	25.2 ± 6.0*^&^
OVA + H-ASD + LPS	5	3.4 ± 0.9*^†§&^	168.6 ± 12.1*^†&^	134.4 ± 52.4*^†&#^	53.9 ± 20.4*^†§&#^	27.5 ± 4.5*^†§&#^	29.7 ± 7.9*^†§&^
TLR4^−/−^ mice							
Control	4	nd	32.5 ± 4.1	nd	5.1 ± 0.3	1.9 ± 0.2	0.1 ± 0.1
LPS	4	nd	35.0 ± 3.9	1.5 ± 0.6	6.0 ± 0.2	2.04 ± 0.26	0.1 ± 0.1
H-ASD	4	nd	224.0 ± 20.8*^†^	63.9 ± 7.3	7.8 ± 0.6	1.57 ± 0.17	5.1 ± 0.1
OVA	5	0.6 ± 0.1	38.3 ± 8.5^§^	2.2 ± 1.1	3.9 ± 0.4	1.2 ± 0.3	nd
OVA + LPS	5	0.5 ± 0.1^β^	42.6 ± 3.6^αβ^	2.9 ± 0.6	5.6 ± 0.6	2.0 ± 0.2^αβ^	nd
OVA + H-ASD + LPS	5	0.6 ± 0.3^β^	80.0 ± 12.5^§αβ^	43.5 ± 12.2^αβ^	8.3 ± 1.3^αβ^	4.3 ± 1.7^αβ^	2.4 ± 1.2^αβ^
MyD88^−/−^ mice							
Control	4	nd	10.9 ± 1.0	nd	2.5 ± 0.3	1.0 ± 0.2	0.9 ± 0.1
LPS	4	nd	10.1 ± 0.6	0.7 ± 0.7	2.9 ± 0.3	1.04 ± 0.23	0.6 ± 0.1
H-ASD	4	0.1 ± 0.1	38.8 ± 12.0	33.3 ± 9.7	4.1 ± 0.6	1.59 ± 0.23	2.6 ± 0.3
OVA	5	0.8 ± 0.1	10.2 ± 0.8	0.9 ± 0.6	5.4 ± 0.8	0.7 ± 0.1	nd
OVA + LPS	5	0.7 ± 0.0^β^	13.0 ± 1.6^αβ^	5.3 ± 1.0	6.2 ± 0.5	2.1 ± 1.0^αβ^	nd^αβ^
OVA + H-ASD + LPS	5	1.0 ± 0.1^β^	24.5 ± 1.6^αβγ^	57.9 ± 9.7	6.9 ± 1.2^αβ^	2.3 ± 0.4^αβ^	nd^αβ^

Wild type (WT), toll like receptor (TLR)2^−/−^, TLR4^−/−^, myeloid differentiation primary response gene 88 (MyD88)^−/−^, four kinds of mouse were divided into six groups with 4–5 (n) which were treated intratracheally with saline (Control), lipopolysaccharide (LPS), heated-Asian sand dust (H-ASD), ovalbumin (OVA), OVA + LPS, OVA + H-ASD + LPS. Four times at 2-week intervals. All values were expressed as mean ± SEM

*nd* not detected

*p < 0.05 vs. control; ^†^*p* < 0.05 vs. LPS; ^§^*p* < 0.05 vs. H-ASD; ^&^*p* < 0.05 vs. OVA; ^#^*p* < 0.05 vs. OVA + LPS; ^α^*p* < 0.05 vs. WT within the same group; ^β^*p* < 0.05 vs. TLR2^−/−^ within the same group; ^γ^*p* < 0.05 vs. TLR4^−/−^ within the same group

### Enhancement of OVA-specific IgE and IgG1 by H-ASD and LPS

**Fig. 5:**
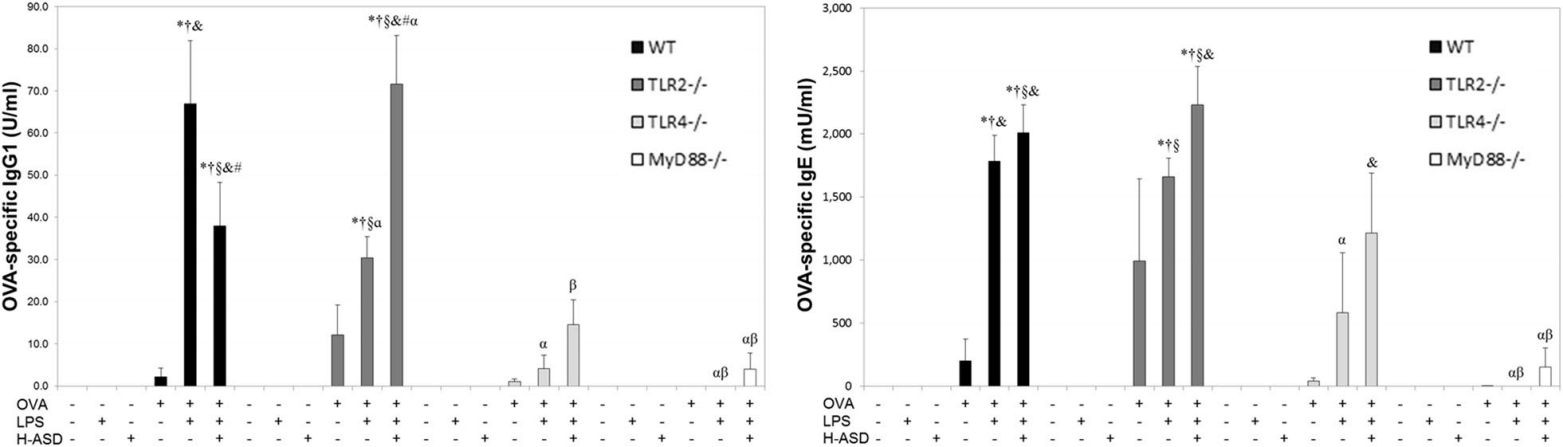
Effects of testing samples on IgE and IgG1 production in serum. According to the manufacturer’s protocol, 1 U of the anti-OVA IgG1 is defined as 160 ng of the antibody. Wild type (WT), toll like receptor (TLR)2^−/−^, TLR4^−/−^, myeloid differentiation primary response gene 88 (MyD88)^−/−^, four kinds of mouse were divided into six groups which were treated intratracheally with saline (control), LPS (50 ng LPS); heated-Asian sand dust (H-ASD) (0.1 mg H-ASD); ovalbumin (OVA), OVA + lipopolysaccharide (LPS), OVA + H-ASD + LPS. Four times at 2-week intervals. All values were expressed as mean ± SEM. *P < 0.05 vs. control; ^†^*P* < 0.05 vs. LPS; ^§^*P* < 0.05 vs. H-ASD; ^&^*P* < 0.05 vs. OVA; ^#^*P* < 0.05 vs. OVA + LPS; ^α^*P* < 0.05 vs. WT within the same group; ^β^*P* < 0.05 vs. TLR2^−/−^ within the same group; ^γ^*P* < 0.05 vs. TLR4^−/−^ within the same group

Figure 5 reveals the effects of the indicated treatments on the serum levels of OVA-specific IgE and IgG1 in mice. Notably, both IgE and IgG1 were not detected in the control group of WT mice. Trace levels of IgE and IgG1 were observed in OVA group, and the levels were significantly increased in OVA + LPS and OVA + H-ASD + LPS groups. The changing pattern of IgE and IgG1 levels in TLR2^−/−^ mice was similar to that in WT mice, whereas the levels of IgE and IgG1 were decreased in TLR4^−/−^ mice treated with OVA + H-ASD + LPS compared to WT and TLR2^−/−^ mice with the same treatment. In MyD88^−/−^ mice, IgG1 was detected only in OVA + H-ASD + LPS group, while IgE was detected in OVA and OVA + H-ASD + LPS groups. Furthermore, the levels of IgG1 and IgE in MyD88^−/−^ mice treated with OVA + H-ASD + LPS were significantly decreased compared to those in WT and TLR2^−/−^ mice with the same treatment.

## Discussion

Co-exposure of 50 ng LPS and H-ASD could trigger the exacerbation of allergic inflammation. Although LPS has already been shown to exacerbate allergies in previous studies, few studies have reported the enhancing effects of both LPS and PM on airway inflammation in mice and the role of TLRs in this process. The results of our study indicate that the murine lung eosinophilia exacerbated by co-exposure of LPS and H-ASD is not only mediated by TLR4/MyD88 signaling pathway, but also TLR4-independent signaling pathways.

Firstly, in the presence of OVA, LPS increased the contents of eosinophils, lymphocytes, IL-5, IL-13 and eotaxin in BALF, as well as enhanced goblet cell proliferation in the bronchial epithelium and induced the recruitment of eosinophils and lymphocytes into the airway submucosa of WT mice. Moreover, in the presence of OVA + H-ASD, the effect of LPS becomes more significant. Eosinophil accumulation is a distinctive feature of lung allergic diseases, including asthma [17]. IL-5 generated from Th2 cells attracts and activates eosinophils, leading to tissue destruction in allergic asthma [18]. IL-13 and eotaxin have been demonstrated to promote eosinophilia [19–21]. Additionally, IL-13 has been shown to increase mucous secretion and mucous cell production, such as goblet cells, in the bronchial epithelium [22]. Therefore, eosinophilic airway inflammation exacerbated by the co-exposure to LPS and H-ASD may be mediated by both cytokines and chemokines. Furthermore, a recent study demonstrates that iron and oxidative stress are at least partly involved in lung eosinophilia exacerbation caused by LPS + H-ASD [23].

Secondly, the lung eosinophilia was more severe in TLR2^−/−^ mice than in TLR4^−/−^ mice, probably due to the fact that TLR2 is not involved in the immune response to LPS [24]. Considering that TLR2^−/−^ mice expressed TLR4 protein, LPS can activate Th2 responses in the mice via TLR4/MyD88 pathway. However, in MyD88^−/−^ mice, a mild eosinophilic inflammation was observed when exposed to with OVA + H-ASD + LPS, which may be mediated by MyD88-independent pathway. Through TLR4, MyD88-independent pathway (TLR4/TRIF pathway) can confer on dendritic cells the ability to support Th2 immune responses [25]. Upon recognition of LPS, TLR4/MyD88-dependent pathway may lead to an early-phase activation of mitogen-activated protein kinases (MAPKs) and nuclear factor-κB (NF-κB) kinases, whereas MyD88-independent pathway contributes to a late-phase activation of MAPKs and NF-κB kinases. In addition, MyD88-independent pathway can also induce the expression of inflammatory cytokines, even though the main role of this pathway is to activate the expression of Type I IFNs [26]. Therefore, in this study, a mild eosinophilic inflammation may have occurred in the MyD88^−/−^ mice exposed to OVA + H-ASD + LPS. In the present study, We also observed a slightly reduction in inflammation of the TLR2^−/−^ mice compared to the WT mice, previous report suggested that hydrogen peroxide, which generated during inflammation, stimulates TLR2 to produce cytokines [16, 27], to that extent, TLR2^−/−^ mice may cause inflammation suppression.

It is worth noting that although the number of eosinophils in BALF is relatively low in TLR4^−/−^ mice, the expression levels of IL-5, IL-13 and eotaxin are somewhat increased by OVA + H-ASD + LPS. A previous study has suggested that LPS triggers caspase-11 activation through noncanonical inflammasome pathway, resulting in the caspase-11, caspase-1, pyrin domain-containing 3 (NLRP3), and apoptosis-associated speck-like protein (ASC)-dependent secretion of IL-1β and IL-18, and TLR4 is dispensable for intracellular LPS to activate this pathway [28]. Besides, NLRP3 act as a key transcription factor in Th2 differentiation [29], IL-1β has been thought to promote a combined Th2- and Th17-type sensitization [30], and IL-18 has been shown to induce IgE production [31]. Therefore, TLR4-independent signaling pathways, such as noncanonical inflammasome pathway, may be involved in LPS-activated Th2 responses to OVA.

As consistent with our previous study [8], in WT mice, OVA + LPS increased the contents of neutrophils, IL-1β, IL-6, IL-12, IL-17A, KC, MCP-1, and TNF-α in BALF, while OVA + H-ASD + LPS exhibited the highest levels of all proteins and neutrophils. Acute exacerbation of severe asthma is associated with the recruitment and activation of neutrophils in the airways [32, 33]. The upregulated expression levels of IL-1β, IL-6, IL-12, IL-17A, KC, MCP-1 and TNF-α may contribute to airway neutrophilia and asthma exacerbation [34–40]. The enhancement of H-ASD on LPS-induced inflammation may react through TLR4/MyD88-dependent pathway, given that the results of a recent study indicate that H-ASD-induced NF-κB activation affects the expression levels of inflammatory proteins through TLR4/MyD88-dependent pathway [41]. Besides, the LPS-induced overexpression of these cytokines and chemokines may be primarily mediated by MAPKs and NF-κB kinases following TLR4 activation [26], and the LPS-induced MAPKs activation can also be regulated, at least in part, by reactive oxygen species (ROS) [42]. In addition, Triggering Receptors Expressed on Myeloid Cells (TREM)-1 signaling also amplifies inflammatory responses to LPS by inducing the secretion of TNF-α and IL-1β [43]. Taken together, in addition to TLR4-dependent pathway, we speculate that TLR4-independent pathway may be involved in the lung inflammation triggered by OVA + H-ASD + LPS.

Furthermore, in the presence of H-ASD and OVA, LPS increased the production of OVA-specific IgE and IgG1 in serum, which is consistent with our previous study [8]. Allergen-specific IgE is a very important biomarker for allergic asthma, and the binding of allergens to allergen-specific IgE activates the release of proinflammatory mediators (e.g., histamine, prostaglandins, leukotrienes), resulting in allergic symptoms [44, 45]. Moreover, allergen-specific IgG1 typically result in cell degranulation through an Fcγ RII receptor on the surface of eosinophils [46]. Therefore, these antibodies can play an important role in the aggravation of lung inflammation induced by OVA + H-ASD + LPS. With respect to the mechanisms underlying the effects of LPS on antibody production, it is well accepted that IL-13 can stimulate B cells and subsequently produce IgE [47]. Indeed, a recent study has demonstrated that TLR4-MyD88-leukotriene B4 receptor (BLT)2-NADPH Oxidase (Nox) 1-ROS-NF-κB cascade contributes to the production of IL-13 by stimulating LPS/TLR4 in mast cells [48].

The hygiene hypothesis of LPS suggested that LPS suppresses allergies. However, previous studies also suggested that LPS exposure is a significant risk factor for increased asthma prevalence [1, 2]. In addition, Compared with the testing sample used in our experiments, there are more pollutants in the environment and their effects on the body are more complex, so when there are LPS, ASD and other substances in the environment, their comprehensive effects on the body may be different from the results of this experiment.

## Conclusions

In summary, our results suggest that LPS and H-ASD activate OVA-induced Th2 response in mice, and exacerbate lung eosinophilia via TLR4/MyD88, TLR4/TRIF and other TLR4-independent pathways. And epigenetics strongly involved in environmental effects on allergy [49, 50]. However, the specific mechanism awaits further investigation. The findings of this study indicate that several environmental conditions (i.e. exposure to a certain concentration of particulate matter and higher levels of LPS) appear to increase the risk of asthma through a wide panel of signaling pathways. Now some good inhibitors of TLR4 have been developed, such as TAK-242 and C34, further investigation of these inhibitors on lung eosinophilia may provide new ideas for the prevention and treatment of human asthma.

## Data Availability

All data generated or analysed during this study are included in this published article.

## Abbreviations

ASC: apoptosis-associated speck-like protein
ASD: Asian sand dust
LPS: lipopolysaccharide
BALF: bronchoalveolar lavage fluid
BMDM: bone marrow-derived macrophages
ELISA: enzyme-linked immunosorbent assays
H-ASD: heated Asian sand dust
IFN-γ: interferon-γ
IL: interleukin
KC: keratinocyte chemoattractant
MAPKs: mitogen-activated protein kinases
MCP-1: monocyte chemotactic protein-1
MCP-3: monocyte chemotactic protein-3
MyD88: myeloid differentiation factor 88
NLRP3: pyrin domain-containing 3
NF-κB: nuclear factor-κB
PRRs: pattern recognition receptors
TLRs: toll-like receptors
TNF-α: tumor necrosis factor-α
TREM: triggering receptor expressed on myeloid cells
TRIF: TIR-domain-containing adapter-inducing interferon-β
